# Improvement of consolidation properties of clay soil using fine-grained construction and demolition waste

**DOI:** 10.1016/j.heliyon.2022.e11029

**Published:** 2022-10-11

**Authors:** Shriful Islam, Junaidul Islam, Nur Md. Robiul Hoque

**Affiliations:** a Department of Civil and Environmental Engineering, Shahjalal University of Science and Technology, Sylhet 3114, Bangladesh

**Keywords:** Construction and demolition waste, Clay soil, Consolidation, Soil improvement, Permeability

## Abstract

As urbanization spreads rapidly, more structures are being built, and more construction and demolition waste (CDW) is produced, occupying about 36–40% of the total solid waste generation in the world; hence, CDW has become a burden nowadays. Moreover, the construction of low-rise buildings on weak soil is always challenging and costly due to the soil's high compressibility and low bearing capacity. Sand or other granular materials are commonly used to improve the compressibility behavior and associated settlement, drainage, and shear strength of weak soil. The massive use of natural sand for construction purposes of different civil engineering structures have lessened their reserves in recent times, increasing their price and destroying the balance in the environment. Among the several methods of improving soil, this research uses fine-grained CDW to improve the geotechnical behavior of weak soil under study. The main objective of this research is to observe the changes in soil properties after mixing with CDW. Recycled waste mortar powder has been selected as CDW mixed in different percentages in the soil. In addition, CDW powder was inserted into soil mass as a circular powder column in triangular and square grid patterns as an alternative to the sand column. CDW in the soil samples improved consolidation settlement, and reduced settlement time and compression index. Increments in the pre-consolidation pressure, consolidation rate, and permeability of the clay-CDW mixtures were also remarkable. Soil improvement through reusing CDW is a sustainable way to solve problems in solid waste management and the soft soil settlement issue under a shallow foundation, ultimately reducing the environmental footprints, saving natural resources, and supporting the circular economy concept.

## 1 Introduction

Before the construction of an engineering project, the existing project area or ground must be checked to see whether it can bear the structural load of the building or structures. Buildings, roads, and other structures often fail due to consolidation settlement of the foundation built on weak soil. In response to the structural load and foundation failures, various ways have been evolved to overcome or mitigate the consequences of poor sub-soil conditions (West, 2015). Rather than a lack of adequate engineering solutions, the destruction caused by weak soils is due to a failure to identify the severity of the soil's settlement or expansion at the start of the design process (Firoozi et al., 2017). As a result, improving soft soil is necessary for construction and development in an acceptable way (Bo et al., 2005; R. Islam et al., 2019). Soil improvement aims to increase strength, bearing capacity, load resistance, and stability, and reduce permeability, compaction tendency, and settlement, which are necessary for successful subsurface performance (Winterkorn and Pamukcu, 1991). The geotechnical engineers face the issue of providing appropriate foundation performance at a cheap cost as a growing percentage of the construction occurs on the weak ground (Charles, 2002). In case of unfavorable subsoil conditions, the geotechnical engineer should constantly examine measures to improve the soil qualities at the site (Holtz et al., 2001). Because of insufficient shear strength and high compressibility, ground improvement activities for soft soils are more complicated than for regular subgrade soils (Bo et al., 2005).

Geotechnical engineers and specialists (Nicholson, 2015; Makusa, 2013; Gaafer et al., 2015; Han, 2015; Russ, 2012; Islam et al., 2018; Verma et al., 2021) have narrated different methods for ground improvement as well as soil stabilization such as consolidation, dynamic compaction, prefabricated vertical drain (PVD), grouting, mixing admixture or additive materials with soil, soil replacement, etc. Among the several methods of improving ground, this research focuses on using fine-grained construction and demolition waste (CDW). The main goal of this research is to improve the geotechnical behavior of soil in presence of CDW, which can potentially reduce the excessive use of sand or other natural resources.

### 1.1 Construction and demolition waste (CDW)

Construction and demolition waste (CDW) is produced during the construction of any civil engineering structure or demolition project. Concrete, plaster, metal, wood, plastics, and other complex and non-biodegradable substances make up the most of it (Poon et al., 2001; Jayatheja et al., 2021a). Construction and demolition waste are diverse and influenced by the country's socio-economic status and local engineering practices (Asprone et al., 2015). CDW generation is now creating a very alarming situation and occupying about 30–40% of the total solid waste produced in the world (Alsheyab, 2022; Akhtar and Sarmah, 2018), which is two and four times the total household trash produced in the United States and Europe, correspondingly (Sáez and Osmani, 2019). Around 10 billion tons of CDW are produced worldwide yearly (Wang et al., 2019), with 2 billion tons generated in China (Zheng et al., 2017). Though a small amount of this waste is recycled (up to 10%), the maximum portion of this vast amount of CDW is just dumped without management (Ragossnig, 2020; Hossain et al., 2017; Bovea and Powell, 2016; Ding and Xiao, 2014; Menegaki and Damigos, 2018). In comparison to many industrialized and developing countries, Bangladesh produces significantly more waste from CDW due to the absence of awareness, a lack of law enforcement and a lack of public sensitization (R. Islam et al., 2019).

### 1.2 Environmental impact of CDW

CDW has become a significant concern because of its management cost and its negative impact on the environment (Li et al., 2013; Tafesse et al., 2022). CDW has environmental consequences, including soil contamination, water pollution, soil fertility losses, climate change, the greenhouse effect, public health, and reducing public space (Wu et al., 2015). It is well-known that demolished waste also contributes to the global warming issue, which contributes to increasing climate extremes, such as heatwaves and poor air quality (Marzouk and Azab, 2014). Again, there are some global concerns during the waste treatment process, including pollution of overland water and groundwater because CDW contains different components. The management methods include various inputs and the discharge of numerous contaminants (Wu et al., 2021). In environmental, social, and economic sectors, CDWs are becoming extremely problematic (Marzouk and Azab, 2014). The environmental benefits of CDW recycling are heavily discussed and dependent on local conditions. On the other hand, life cycle thinking provides a comprehensive view of an activity's impact on the environment. It has been used to weigh the benefits and drawbacks of recycling CDW in numerous nations (Jain et al., 2020). Although CDWs are generally not considered hazardous, their accumulation may cause major environmental issues (Simion et al., 2013).

### 1.3 Soil improvement with CDW

Different researchers (Pourkhorshidi et al., 2020; Zhang et al., 2019; Kerni et al., 2015; Jayakody et al., 2019; Jayatheja et al., 2021) found that processed and selected CDW is an excellent option for improving the geotechnical behavior of soil having lower strength and high compressibility for applying in pavement constructions, backfilling materials and structures with lower load, ensuring the recycling and reuse of CDW (Rahman et al., 2013, 2014; Arulrajah et al., 2019; Henzinger and Heyer, 2018). Dobrescu and Calarasu (2020) emphasizes the significance of the soil improvement capability of CDW when mixed with soil. Recycled fine aggregates of concrete, brick and mortar from CDW with proper size and proper mixing ratio with soil give a satisfactory result in improving different geotechnical properties of the existing soil (Mohammadinia et al., 2018; Varaprasad et al., 2019). In poor clayey soil, using an optimal amount of CDW improves the unconfined compressive strength, CBR, and permeability. The increased secant modulus and the regression analysis performed for various tests revealed that laboratory results and anticipated values were upgraded as CDW is added to natural soil (Oskooei et al., 2020; Sharma and Sharma, 2020). Using recycled CDW as a compaction pile alternative in foundation construction for soil improvement has a good prospect (Farias et al., 2012). The type of fine-grained soil largely determined the oedometer features, particularly the swelling and consolidation of CDW-clay mixes (Mohialdeen et al., 2020). In addition to necessity, CDW aggregates can also be stabilized by fly ash, lime kiln dust, and cement kiln dust (Mohammadinia et al., 2018a, 2018b). According to Farias et al. (2012), the civil construction industry employs CDW to help it adapt to the environment and abide to sustainable development principles such as recycling, illegal dumping, and pollution reduction.

Recently, improving poor soil using CDW has become an interesting topic among researchers (Yuan and Shen, 2011; Arisha et al., 2016; Duan et al., 2020; Bagriacik and Mahmutluoglu, 2020). Using CDW in ground improvement work can solve geotechnical engineering and environmental threats. As a massive amount of CDW becomes a burden issue throughout the world (Ding and Xiao, 2014; Dahlbo et al., 2015), sustainable management and recycling of CDW and using it as engineering materials can make a satisfactory change to ensure a sustainable environment for the future (Lukiantchuki et al., 2019; Merino et al., 2010). As a result, it is evident that applying CDW to improve poor clayey soil will resolve the issue of its disposal, which will also save the money required for CDW management and protect our environment (Sharma and Sharma, 2020).

Many researches showed the use of CDW in improving the geotechnical behavior of soil. Jayatheja et al. (2021) used coarse CDW aggregates with poor sand as a backfilling material, while Zhang et al. (2020) used them as filling materials in highway subgrade. Arulrajah et al. (2017) mixed different types of CDW aggregates with plastic waste in various portions to increase the soil strength and stiffness, while Arulrajah et al. (2020) described the use of CDW aggregates with plastic waste as railway capping materials. Jayatheja et al. (2017) narrated the performance of CDW partially replaced cohesionless soil, while Suluguru et al. (2018) characterized the CDW materials for replacing subgrade pavement. Mohammadinia et al. (2014) treated CDW using cement and applied them to pavement subbase construction, while Mohammadinia et al. (2019) stabilized CDW coarse aggregates with calcium carbide (CaC_2_) to check flexural fatigue strength. Cardoso et al. (2016) reviewed numerous research works on recycling CDW for using in pavement construction, and their physical and mechanical behavior along with resilient modulus, bearing capacity, and hydraulic characteristics have been presented.

To add to the above findings, this research intended to use fine-grained powder of recycled mortar CDW to improve the consolidation behavior of clay soil as well as observing compaction characteristics and Atterberg limits. Mortar CDW was powdered as close to the clay particle sizes in the range of 0.002 mm–0.06 mm so that it reaches the sizes of natural soil in the site, where most of the researchers used CDW in coarser size and implemented with other waste. In this research, fine-grained CDW was mixed clayey soil uniformly to improve the consolidation behavior of clayey soil. Also, circular columns of CDW powder are placed in soil mass in triangular and square grid patterns since soil replacing with additive for large-scale soil stabilization is difficult in the actual field. As uniform mixing of soil-CDW in the larger areas needs well-equipped technology that might be costly, users may lose interest in soil replacement in uniform mixing. In this case, powder CDW column can be inserted in soil mass is an easy way like a sand column. Hence, rather than other literature showing improvement of weak soil using a uniform mix of CDW and other additives, this research shows the potential use of CDW powder column in soil mass to make the recycling of CDW easily applicable in all possible scale of conditions, bringing a new term waste powder column for soil improvement.

## 2 Materials and methods

### 2.1 Materials

**Figure 1:**
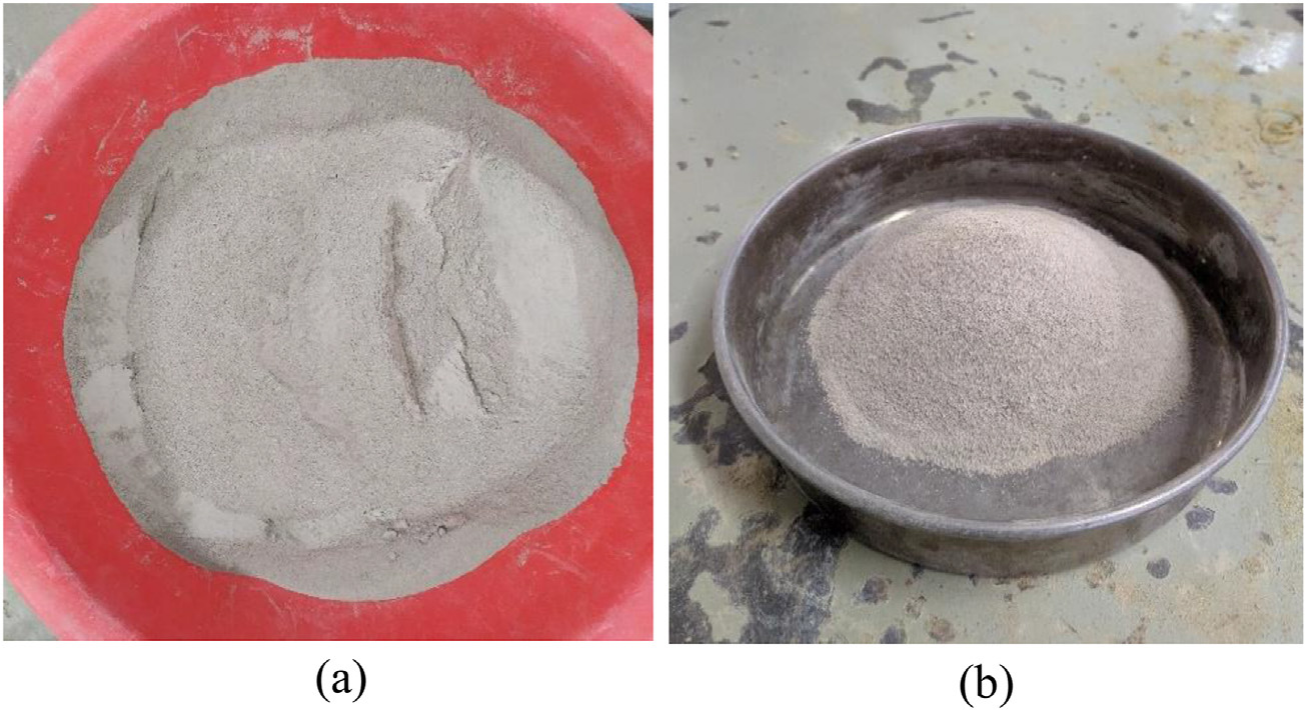
Used materials: (a) clay soil, and (b) waste mortar powder.

The clayey soil was collected from a site of a proposed three-storied building in Sylhet City, Bangladesh. Collected clay soil samples were oven-dried at 60 °C temperature to prevent the combustion of the carbonaceous matter, if any and then crushed using a wooden hammer, and all the portion of the crushed soil passed through the sieve of size 0.002 mm. Mortar construction and demolition waste (CDW) was collected from the site of a local demolished building and appropriately cleaned to remove the surface color, broken brick chips, and other impurities. The mortar CDW was cleaned with deionized water and dried in the oven at a 60 °C temperature until the moisture was completely removed. The CDW mortar was crushed using mortar and pestle and was sieved to achieve particle sizes in the range of 0.002 mm–0.06 mm following ASTM D422 (2007). The specific gravity of the used clay soil and mortar aggregates were found to be 2.65 and 2.72, respectively, in specific gravity tests followed by ASTM D854 (2014). Figure 1 shows the crushed clay soil and fine-grained CDW used in this research.

### 2.2 Methods

#### 2.2.1 Mixing proportion and specimen preparation

For comparing the results of different tests, CDW powder was mixed with soil samples in the ratios of 0% (no CDW), 10%, 20%, 30%, and 40%. Soil specimens and soil-CDW mixtures were prepared following ASTM STP 599 (1976).

#### 2.2.2 Atterberg limit test

Atterberg limit tests were carried out to observe the liquid limit (LL), plastic limit (PL), and plasticity index (PI) of the original soil sample and CDW-soil mixtures following ASTM D4318 (2017).

The liquid limit of the soil sample was determined at the water content when a portion of clay inside a standard cup was cut by a trench of standard diameters mixed together for 13 mm at the bottom of the gap, due to shocks applied to the cup ASTM D4318 (2017). The plastic limit of a soil sample is the water content at which a 3.175-mm thread of sample was just crumbled when it was rolled on plain glass. At this water content, the paste of a geomaterial converts from a semi-solid state to a plastic state.

#### 2.2.3 Compaction test

Laboratory compaction tests were carried out on original soil and soil-CDW mixtures to determine the soil's optimum moisture content (OMC) and maximum dry density (MDD). In this research, standard proctor test was implemented in 600 Kn-m/m^3^ effort in a mold of 6 inches (152.4 mm) diameter, and the soil was compacted into three layers with 56 blows in each layer following ASTM D698 (2007). Initially, moisture content started from 8.5% for the original soil sample, and the wet density was measured in each step until it became maximum and then decreased after increasing water content. After measuring wet density for each soil sample and soil-CDW mixtures for different water content, a portion of each sample was taken for oven-dry to determine the moisture content present in the soil. Then the dry density of each sample in different moisture content was determined using Eq. (1).

(1) $$\rho_{d}=\frac{\rho }{1+w}$$

Here, $\rho_{d}$ = dry density of soil, ρ = moist density of soil, and *w =* moisture content*.*

#### 2.2.4 Consolidation test

**Figure 2:**
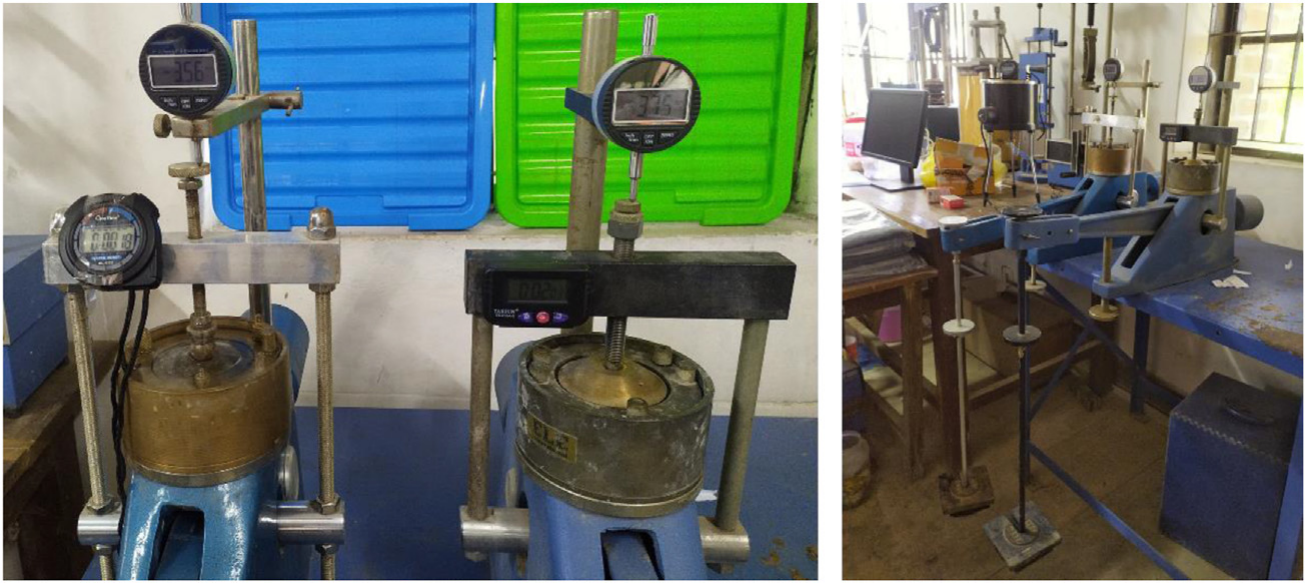
Consolidation test set up used in this research.

The consolidation test was operated to examine the settlement behavior of the soil sample and soil-CDW mixtures. Figure 2 shows two-consolidation test setups that were used in this research. The test procedure follows the ASTM D2435M (2011). Consolidation settlement in each step of loading has been analyzed. Coefficient of consolidation (*c*_*v*_), compression index (*C*_*c*_), coefficient of permeability (*k*), and pre-consolidation pressure (*σ’*_*pc*_) were determined for all the tests carried out to investigate the effect of CDW in the weak soil. In this research, incremental load was applied on the sample in 6 steps, producing stresses of 6.11 kPa, 12.22 kPa, 24.45 kPa, 48.9 kPa, 97.8 kPa and 195.6 kPa. The displacement dial readings were taken at different time intervals up to 24 h to achieve the complete primary consolidation of the sample in each loading step.

The height of solids *(H*_*S*_*)* present in the consolidation ring is calculated using Eq. (2) (Das, 2010).

(2) $$H_{s}=\frac{W_{s}}{G_{s}\gamma_{s}A}$$

Where, $H_{s}$ = height of soil solids, *W*_*s*_ = dry weight of soil sample, *G*_*s*_ = specific gravity of soil solids, $\gamma_{s}$ = unit weight of soil solids, and *A* = cross-sectional area of consolidation ring.

Void ratio, *e* has been calculated by employing Eq. (3) from ASTM D422 (2007).

(3) $$e=\frac{H-H_{s}}{H_{s}}$$

Where, *H* = initial height of the soil sample = 20 mm.

The coefficient of consolidation, *c*_*v*_, at each pressure increment was calculated using Eq. (4) according to Casagrande logarithm of time method, which was evolved in Terzaghi's consolidation theory (Das, 2010; Shukla et al., 2009).

(4) $$Cv=\frac{\;0.197\times {\left(H_{dr}\right)}^{2}}{t_{50}}$$

Where, *H*_*dr*_ = drainage depth, and *t*_*50*_ = time taken to reach 50% of primary consolidation settlement.

The compression index, *C*_*c*_, was obtained from the slope of the straight line part of *e* vs.. log σ plot using Eq. (5) (Das, 2010).

(5) $$C_{c}=\frac{e_{1}-e_{2}}{\log_{\sigma 2}-\log_{\sigma 1}}$$

Where, *e*_*1*_ = void ratio at stress 1, *e*_*2*_ = void ratio at stress 2, *σ*_*1*_ = effective at stress 1, *σ*_*2*_ = effective at stress 2.

Coefficient of volume compressibility, *m*_*v*_, was calculated by employing Eq. (6) (Das, 2010).

(6) $$m_{v}=\frac{a_{v}}{{1+e}_{0}}$$

Where, *a*_*v*_ = coefficient of compressibility $=\frac{\Delta e}{\Delta \sigma }$

The coefficient of permeability, *k*, was obtained using Eq. (7) (Das, 2010).

(7) $${k=c}_{v}a_{v}\;\gamma {w/1+e}_{0}$$

Where, *e*_*0*_ = initial void ratio, *Δe* = changes in void ratio, *Δσ* = changes in effective stress.

Pre-consolidation pressure*, σ’*_*pc*_, for all samples was calculated by Casagrande graphical method (Dias and Pierce, 1995).

#### 2.2.5 Powder column

**Figure 3:**
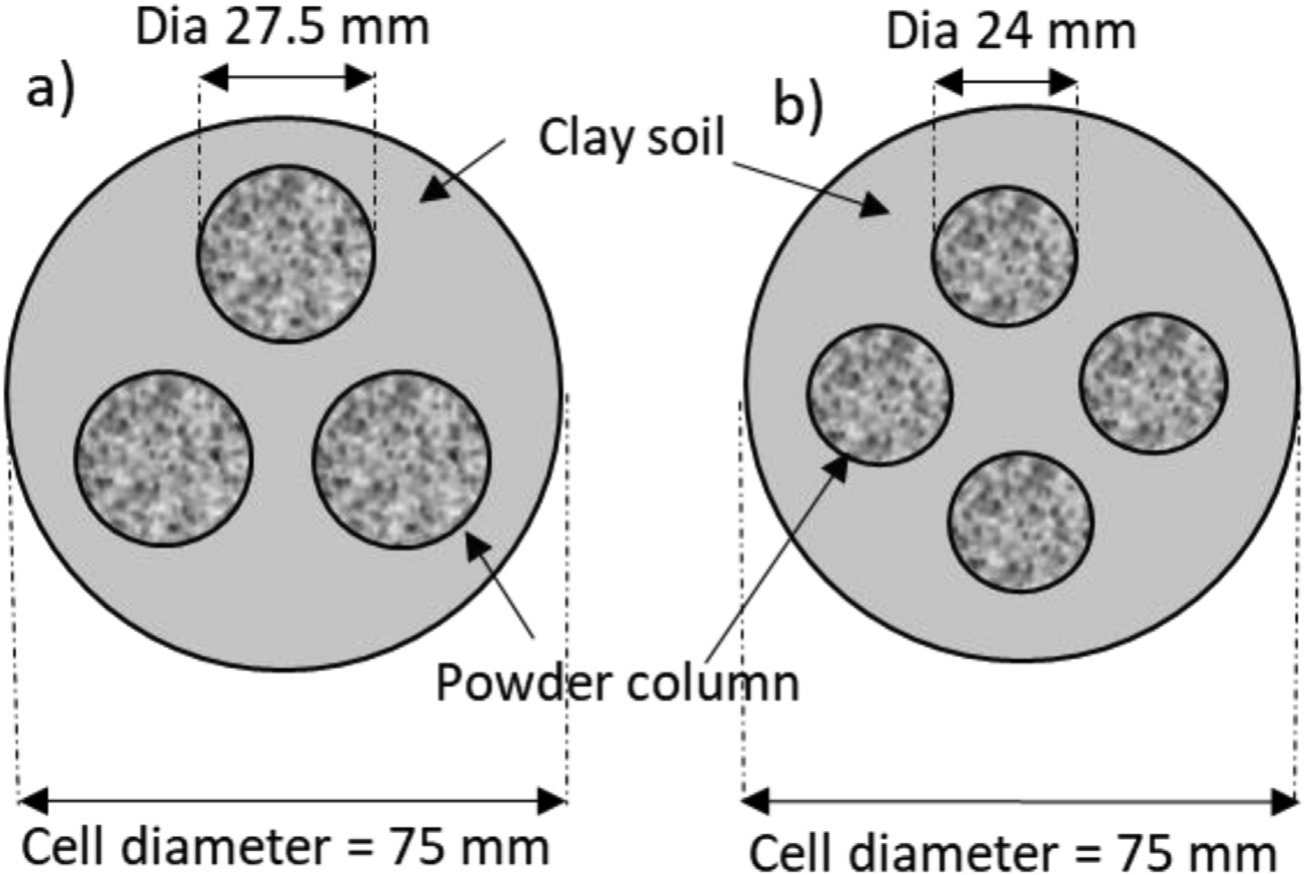
Schematic diagram of circular CDW column in soil mass in (a) Triangular, and (b) Square grid pattern.

The circular CDW powder column was inserted in the soil mass of the consolidation ring in a square and triangular grid pattern. Figure 3 shows the schematic diagram of triangular and square grid patterns. The total volume of the CDW powder column, in both square and triangular grid patterns, was the optimum percentage of CDW, which was 40% of the soil sample. After inserting, consolidation tests were carried out on the samples containing triangular and square pattern CDW columns using the same loading described before. Feng et al. (2015) found CDW column of particle size less than 20 mm fulfilled the requirement for use in roadway pavement. In this research, CDW column is assessed as a fine powder column in the soil mass to check whether it is applicable and shows proper geotechnical engineering properties.

## 3 Results and discussion

### 3.1 Atterberg limit

**Figure 4:**
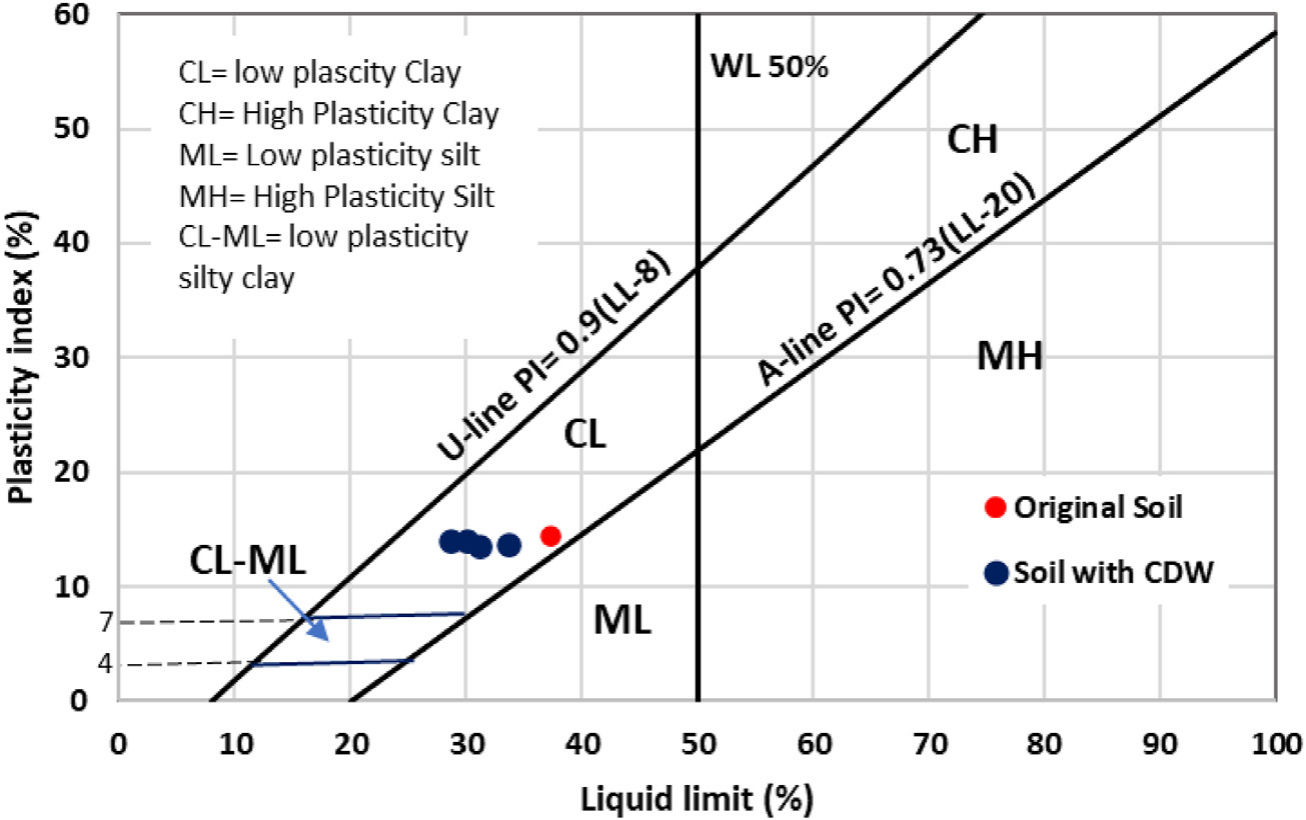
Casagrande Plasticity chart for the soil samples used in this research.

Table 1 presents the test results of the Atterberg limit. As CDW percentages increased in soil mass, LL and PL reduced because the additive CDW particle was mostly granular and non-cohesive materials. The original soil sample provided the maximum LL of 37.39%, PL of 22.96%, and PI of 14.43%. The soil sample with 40% CDW shows the minimum LL of 28.85%, PL of 14.98%, and PI of 13.87%. The Casagrande plasticity chart shown in Figure 4 describes the original soil sample as a low plasticity clay soil positioned just on the A-line. As the soil mixed up with 10% CDW, the position of the sample point moved down left in the plasticity chart as the liquid limit and plasticity index reduced and became 33.78% and 13.55%, respectively. With the addition of 20%, 30%, and 40% CDW, the positions moved further and the liquid limit and plasticity index became 31.25% and 13.28% for 20% CDW, 30.25% and 13.17% for 30% CDW, and 28.85% and 13.87% for 40% CDW, respectively. The samples of original soil and soil-CDW mixture fall in the same zone of low plasticity clay soil but show lower values of LL and PI. In comparison, Abdulnafaa et al. (2021) experienced changes in soil classification of low plasticity clay soil to low plasticity silty soil after adding construction and demolition materials in the soil as the additive materials contained of stone, brick chips and others construction materials. The fine-grained mortar CDW used this research contained sand and cement and hence soil class didn't change rather reduction in Atterberg limit. Due to the non-plastic nature of CDW particles, the addition of CDW in different percentages to clay soil samples lowers the liquid limit and plasticity index, resulting in less swelling of the soil and a lower risk of foundation fractures (Sharma and Sharma, 2019).

**Table 1:** Changes in the liquid limit, plastic limit, and plasticity index due to the addition of CDW in the soil.

Soil types	CDW (%)	Liquid limit (%)	Plastic limit (%)	Plasticity index (%)
Original soil	0	37.39	22.96	14.43
10% CDW-soil	10	33.78	20.23	13.55
20% CDW-soil	20	31.25	17.89	13.36
30% CDW-soil	30	30.25	16.35	13.28
40% CDW-soil	40	28.85	14.98	13.17

### 3.2 Compaction test

**Figure 5:**
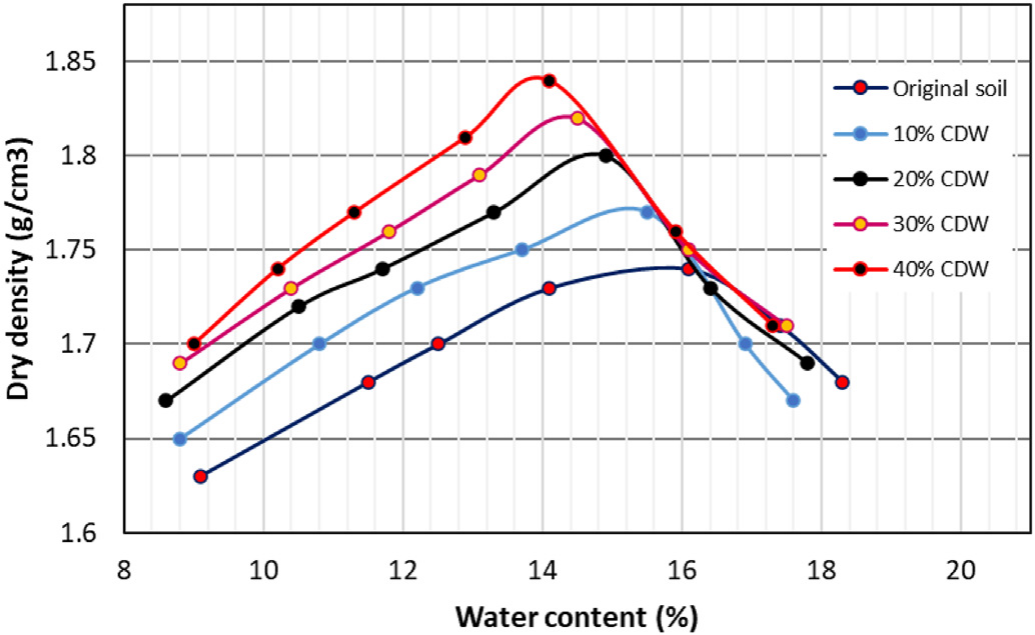
Compaction curve for different soil sample.

**Figure 6:**
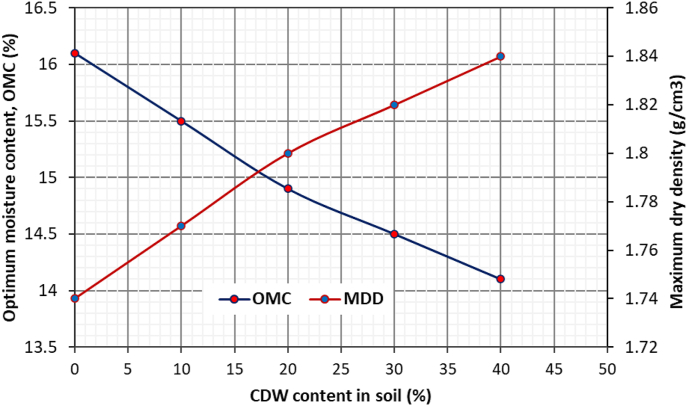
Compaction characteristics of soil samples.

Figure 5 shows the dry density vs. water content plot of the compaction test for the original soil and soil-CDW mixtures, which are also presented in detail in Table 2. The original soil sample has an optimum moisture content (OMC) of 16.1% and a maximum dry density (MDD) of 1.74 g/cm^3^. As CDW content increased in the soil sample, there was a decrease in OMC and increase in MDD. As seen in Figure 5, the compaction curve changes their peak with the addition of CDW in soil. As the CDW content increased in the soil sample, the optimum moisture content decreased because the non-cohesive CDW powder absorbed less water than natural soil particles and maximum dry density increased because CDW powder has more sands and greater specific gravity than clayey soil (Abdulnafaa et al., 2021). The changes in optimum moisture content and maximum dry density of soil sample due to CDW percentages are shown in Figure 6. Different researchers have justified these changes in OMC and MDD of soil after adding CDW. Deng et al. (2021) observed 50% CDW mixed with silty soil has the maximum dry density, UCS and CBR value. Mohammadinia et al. (2018a) experienced reduced OMC and increased MDD of soil after adding CDW with lime kiln dust.

**Table 2:** Compaction test data for different soil sample.

Original soil	Moisture content, w (%)	9.1	11.5	12.5	14.1	16.1	17.4	18.3
	Dry density, DD (g/cm^3^)	1.63	1.68	1.7	1.73	1.74	1.71	1.68
Soil with 10% CDW	Moisture content, w (%)	8.8	10.8	12.2	13.7	15.5	16.9	17.6
	Dry density, DD (g/cm^3^)	1.65	1.7	1.73	1.75	1.77	1.7	1.67
Soil with 20% CDW	Moisture content, w (%)	8.6	10.5	11.7	13.3	14.9	16.4	17.8
	Dry density, DD (g/cm^3^)	1.67	1.72	1.74	1.77	1.8	1.73	1.69
Soil with 30% CDW	Moisture content, w (%)	8.8	10.4	11.8	13.1	14.5	16.1	17.5
	Dry density, DD (g/cm^3^)	1.69	1.73	1.76	1.79	1.82	1.75	1.71
Soil with 40% CDW	Moisture content, w (%)	9	10.2	11.3	12.9	14.1	15.9	17.3
	Dry density, DD (g/cm^3^)	1.7	1.74	1.77	1.81	1.84	1.76	1.71

### 3.3 Consolidation test

#### 3.3.1 Settlement

**Figure 7:**
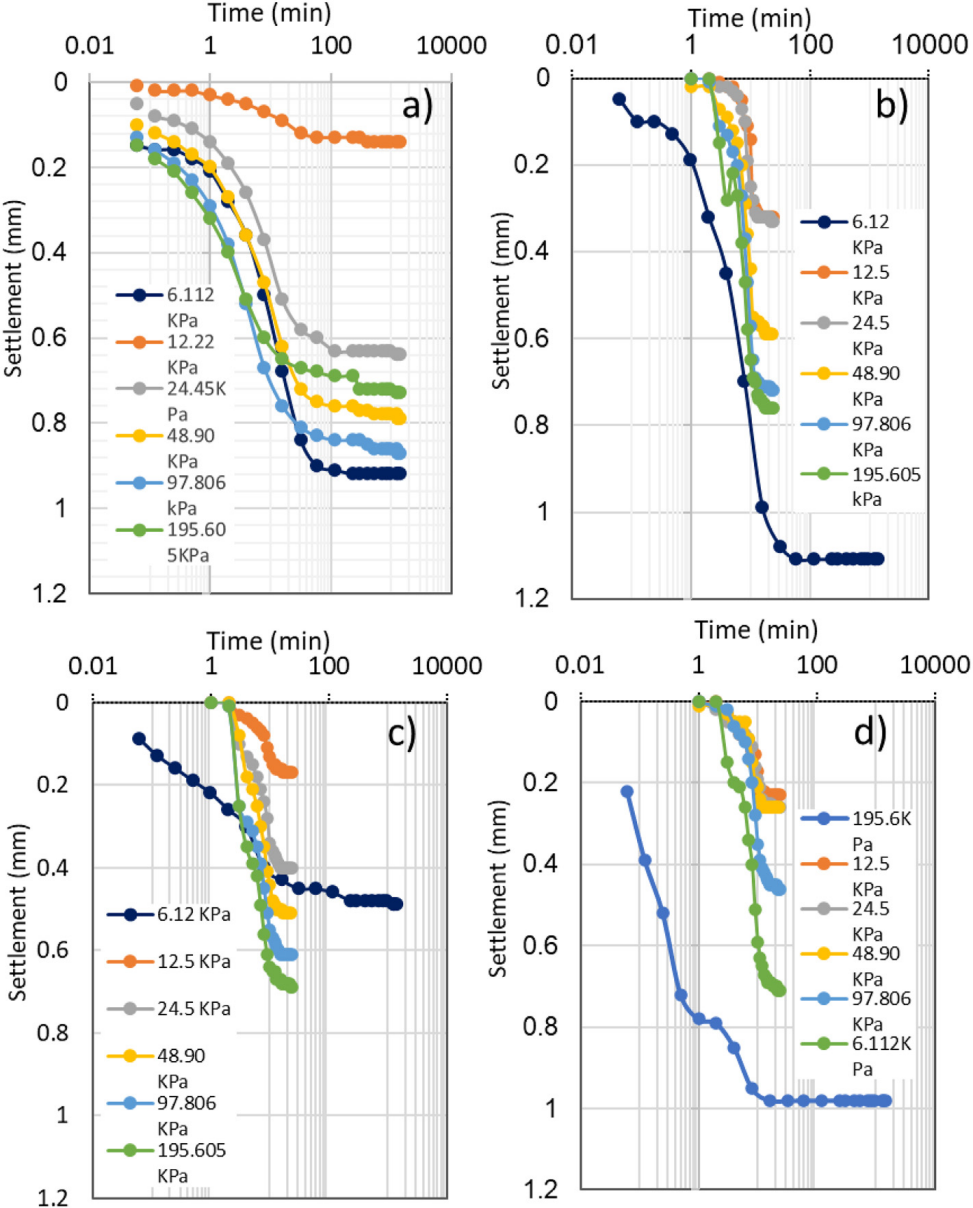
Settlement vs. Time for (a) original soil sample, (b) soil with CDW 40%, (c) soil with circular CDW column in a triangular grid pattern, and (d) with circular CDW column in a square grid pattern.

All the consolidation test results are presented in Table 3. Settlement of the samples against time due to loading in each step has been plotted for the original soil sample, the sample of soil 40% CDW, and samples of CDW column inserted in the soil in square and triangular patterns are presented in Figure 7. The total settlement at the end of the complete test was found as 4.09 mm and 3.56 mm for original soil and soil with 40% CDW, respectively. The soil sample contained 10% CDW, 20% CDW, and 30% CDW has a settlement value of 3.83 mm, 3.75 mm, and 3.73 mm, respectively. The more CDW content in the soil sample, the less settlement found at the end of the loading, while the soil sample containing 40% CDW has the minimum settlement among the variations used in this research. The settlement data changed significantly after inserting the CDW column in triangular and square patterns into the soil sample. Soil samples with CDW columns in triangular and square grid patterns experienced 2.87 mm and 2.90 mm settlements, respectively, which is about 30% less than the original soil sample. Adding CDW powder to the soil mass made the sample more resistant to loading and resulted in a lower settlement (Horpibulsuk et al., 2012; Muktinutalapati et al., 2020). Other studies showed, CDW raised the bearing capacity and strength of the soil in a significant way (Cardoso et al., 2016; Mazhar and GuhaRay, 2020; Naeini et al., 2019). The soil sample with circular CDW columns in triangular and square patterns showed the highest resistance and lowest settlement, though both tests showed similar final settlements. As the CDW columns in the soil in both triangular and square patterns show promising results in reducing the settlement of soil, the soil-CDW mixture could gain higher bearing capacity. Feng et al. (2015) reported that CDW columns of coarse aggregates have satisfactory qualities in reducing soil collapsibility.

**Table 3:** Consolidation test results for different soil samples.

	Pressure (kPa)	void ratio, *e*	*t*_*50*_ (sec)	*c*_*v*_ (cm^2^/s)	*m*_*v*_ (1/kPa)	*k* (m/s)	*C*_*c*_
Original soil sample	0.00	1.15					0.282
	6.11	1.05	455	1.73E-03	7.52E-03	1.28E-08	
	12.22	1.04	450	1.75E-03	1.15E-03	1.97E-09	
	24.45	0.97	372	2.12E-03	2.62E-03	5.44E-09	
	48.90	0.88	370	2.13E-03	1.62E-03	3.38E-09	
	97.09	0.79	332	2.37E-03	9.03E-04	2.10E-09	
	195.61	0.71	266	2.96E-03	3.70E-04	1.08E-09	
	Average		374.17	2.18E-03	2.36E-03	4.46E-09	
Soil with 10% CDW	0.00	1.15					0.2556
	6.11	1.10	480	1.64E-03	4.08E-03	6.57E-09	
	12.22	1.05	357	2.21E-03	3.44E-03	7.44E-09	
	24.45	0.97	480	1.64E-03	2.90E-03	4.67E-09	
	48.90	0.91	510	1.55E-03	1.21E-03	1.83E-09	
	97.81	0.84	120	6.57E-03	6.75E-04	4.35E-09	
	195.61	0.77	117	6.74E-03	3.48E-04	2.30E-09	
	Average		344	3.39E-03	2.11E-03	4.53E-09	
Soil with 20% CDW	0.00	1.15					0.246
	6.11	1.05	470	1.68E-03	7.76E-03	1.28E-08	
	12.22	1.01	445	1.77E-03	3.11E-03	5.40E-09	
	24.45	0.95	312	2.53E-03	2.29E-03	5.67E-09	
	48.90	0.89	253	3.11E-03	1.15E-03	3.50E-09	
	97.81	0.82	168	4.69E-03	6.24E-04	2.87E-09	
	195.61	0.75	108	7.30E-03	3.53E-04	2.52E-09	
	Average		292.67	3.51E-03	2.55E-03	5.46E-09	
Soil with 30% CDW	0.00	1.15					0.223
	6.11	1.07	250	3.15E-03	6.13E-03	1.89E-08	
	12.22	1.04	227	3.47E-03	2.21E-03	7.53E-09	
	24.45	0.98	180	4.38E-03	2.41E-03	1.04E-08	
	48.90	0.90	186	4.24E-03	1.39E-03	5.78E-09	
	97.81	0.82	120	6.57E-03	7.87E-04	5.07E-09	
	195.61	0.75	129	6.11E-03	3.43E-04	2.05E-09	
	Average		182	4.65E-03	2.21E-03	8.29E-09	
Soil with 40% CDW	0.00	1.15					0.210
	6.11	1.03	265	2.97E-03	9.07E-03	2.65E-08	
	12.22	1.00	340	2.32E-03	2.62E-03	5.96E-09	
	24.45	0.96	132	5.97E-03	1.35E-03	7.90E-09	
	48.90	0.90	153	5.15E-03	1.21E-03	6.10E-09	
	97.81	0.82	120	6.57E-03	7.36E-04	4.74E-09	
	195.61	0.74	54	1.46E-02	3.89E-04	5.56E-09	
	Average		177.33	6.26E-03	2.56E-03	9.45E-09	
CDW column in triangular pattern	0.00	1.18					0.182
	6.11	1.10	60	1.27E-02	6.06E-03	7.58E-08	
	12.22	1.08	120	6.16E-03	1.37E-03	8.31E-09	
	24.45	1.04	54	1.33E-02	1.61E-03	2.11E-08	
	48.90	0.98	120	5.70E-03	1.03E-03	5.76E-09	
	97.81	0.92	15	4.29E-02	6.16E-04	2.59E-08	
	195.61	0.84	15	3.99E-02	3.48E-04	1.36E-08	
	Average		64	2.01E-02	1.84E-03	2.51E-08	
CDW column in square pattern	0.00	1.15					0.183
	6.11	1.04	8.4	8.88E-02	8.01E-03	6.98E-07	
	12.22	1.02	9.6	7.30E-02	1.88E-03	1.35E-07	
	24.45	0.99	7.2	9.48E-02	1.06E-03	9.89E-08	
	48.90	0.96	72	9.22E-03	5.32E-04	4.81E-09	
	97.81	0.91	8.4	7.59E-02	4.70E-04	3.50E-08	
	195.61	0.84	14.4	4.15E-02	3.63E-04	1.48E-08	
	Average		20	6.39E-02	6.39E-02	1.64E-07

#### 3.3.2 Coefficient of consolidation

The coefficient of consolidation, *c*_*v,*_ refers to the consolidation rate calculated according to the logarithm of time method with the time required to reach 50% primary consolidation settlement, *t*_*50*_. The time to reach 50% of primary consolidation settlement, *t*_*50*_, was found as 374 s for the original soil sample. After adding 10% CDW to the soil sample, the *t*_*50*_ value was reduced to 344 s. Adding 20% CDW to soil gives a more reduced *t*_*50*_ value of 292 s. Further, 30% CDW and 40% CDW in the soil has a *t*_*50*_ value of 182 s and 177 s, respectively. The soil sample with 40% CDW takes the least time for 50% primary consolidation settlement, *t*_*50*_ (Table 3). For the original soil sample coefficient of consolidation, *c*_*v*_ was found as 2.18E-03 cm^2^/s. The average values of *c*_*v*_ were calculated as 3.39E-03 cm^2^/s, 3.51E-03 cm^2^/s, 4.65E-03 cm^2^/s, and 6.26E-03 cm^2^/s for soil with 10% CDW, soil with 20% CDW, soil with 30% CDW, and soil with 40% CDW, respectively. The more CDW powder content in the soil sample, the greater the consolidation rate found in the experiment. Again, a faster 50% primary consolidation settlement happened in the CDW column inserted soil sample. The soil sample with circular CDW columns in triangular and square grid patterns gave an average *t*_*50*_ of 64 s and 20 s, respectively, which was rapid compared to the soil sample uniformly mixed with CDW. Less *t*_*50*_ value of powder column inserted soil sample resulted in a higher *c*_*v*_, which are 2.01E-02 cm^2^/s and 6.39E-02 cm^2^/s for the sample with CDW column inserted in a triangular and square grid pattern, respectively. Table 3 shows *c*_*v*_ values and other consolidation test results for all the samples. As the CDW content increased in the soil sample, the consolidation rate was affected incrementally. It is because the CDW presented in the clay soil helped to drain the water faster and settled in a faster time. As 40% CDW shows the maximum results in a consolidation rate, 40% CDW is the optimum content. Initially, at the first stage of each loading, the additive CDW fine particle creates voids among the soil, and the voids occupied by water get drained quicker when the load is applied to the soil sample. Again the CDW powder column inserted soil has the minimum value of *t*_*50*_ and the maximum value of *c*_*v*_ (Chu et al., 2012; Horpibulsuk et al., 2012). In a complicated construction site with clay soil, often needed to settle the site as early as possible to avoid the failure of the structure due to consolidation.

#### 3.3.3 Compression index

**Figure 8:**
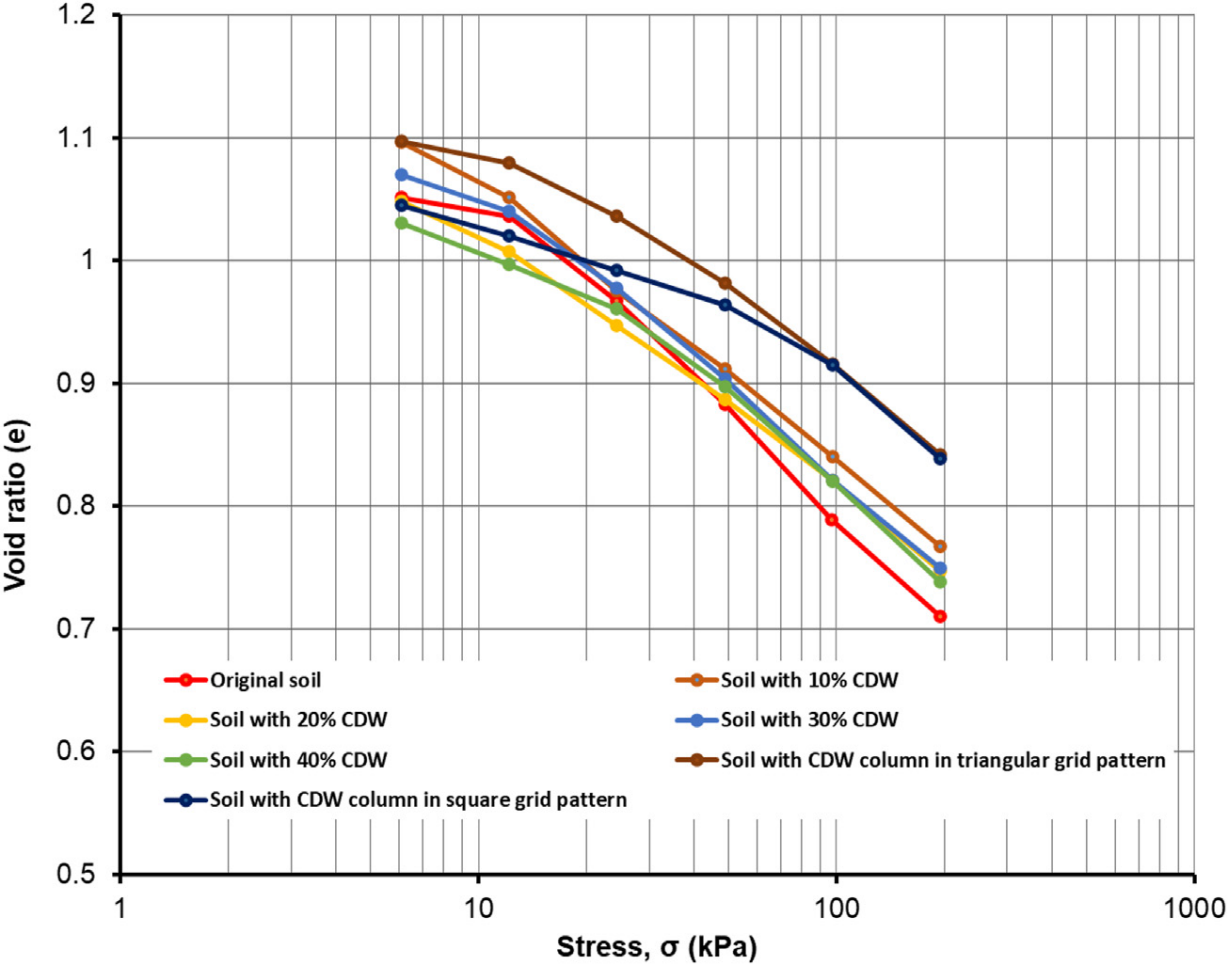
Void ratio vs. effective stress plot for different soil samples.

The compression index, *C*_*c*_*,* was obtained from the void ratio of the soil samples in different loading stages of the consolidation test carried out in this research. In Table 3 the void ratio, *e* in each loading stages are shown for different soil samples. Compression index, *C*_*c*_ for different soil samples was calculated from the slope of *e* vs. log σ graph shown in Figure 8. For the original soil sample, *C*_*c*_ value were found as 0.282. The addition of 10% CDW resulted in a decrease in *C*_*c*_ value is 0.256, then a further increase in CDW percentages as 20%CDW further reduced *C*_*c*_ value and became 0.246, 0.223, and 0.211 for the soil with 20% CDW, soil with 30% CDW and soil with 40% CDW, respectively. Hence, the soil sample mixed with 40% CDW has the minimum compression index value, *C*_*c*_ as 0.21. Also, 40% soil-CDW sample has the lowest settlement among the soil-CDW mixed samples in this research. For this, 40% CDW content becomes the optimum content. The reason of not increasing CDW in the soil-CDW mixture beyond 40% is that the higher percent will change the dominance of clay and affect the soil types. Again, the research targeted to ensure recycling and implementation of a higher amount of CDW as possible, so 40% CDW becomes the threshold amount in this research. Additionally, the soil sample with CDW powder column in a triangular grid-patterned CDW column gave the minimum *C*_*c*_ value as 0.182. In contrast, the soil sample with CDW column in square grid-patterned has a *C*_*c*_ value of 0.183. This significant decrease in the compression index value of CDW powder column inserted soil indicated the lower settlement discussed earlier in Section *3.3.1.* The addition of CDW to the soil sample decreased the compressibility of the soil, which resulted in a lower compression index as well as consolidation settlement (Chu et al., 2012; Sharma and Sharma, 2020). Table 3 shows the values of *C*_*c*_ for all the samples.

#### 3.3.4 Coefficient of permeability

The average coefficient of permeability, *k,* was calculated from the consolidation test results as 4.46E-09 m/s, 4.53E-09 m/s, 5.46E-09 m/s, 8.29E-09 m/s, and 9.454E-09 m/s for the original soil sample, soil with 10% CDW, soil with 20% CDW, soil with 30% CDW, and soil with 40% CDW, respectively (Table 3). It is observed in the results that the values of *k* increased with the increase in CDW in the soil mass that has been supported by Rahman et al. (2013), Rahman et al. (2014) and Abdulnafaa et al. (2021). Abdulnafaa et al. (2021) explained that *k*-value changes because increasing granular and non-cohesive materials in the soil accelerates the passing of water through it. As 40% CDW with the soil gives the maximum *k-*value*,* 40% CDW is the optimal content in the soil mass used in this research. The value of *k* was higher for the sample with the CDW column in the soil mass, giving *k*-values as 2.51E-08 m/s and 1.64E-07 m/s for the sample with CDW column in triangular and square grid patterns, respectively, because the column of CDW creates a drainage path for water to come out of the soil. In the case of a square grid-patterned CDW column set in the consolidation cell, water had to travel a minimum radial distance to the CDW column as the gap between columns was less than that of triangular grid-patterned CDW column set and hence produced higher *k*-value. As CDW column in both triangular and square grid pattern shows higher *k-*value, these can be implemented in road construction, field, and playground construction where permeability is a primary requirement (Rahman et al., 2014; Feng et al., 2015) including soil improvement where shallow foundation needs to be constructed.

**Figure 9:**
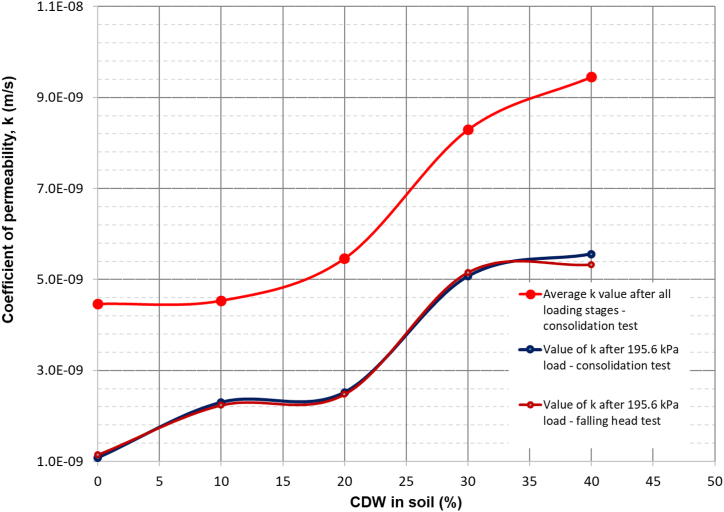
Coefficient of permeability vs. CDW percentages for soil and soil-CDW mixture.

**Figure 10:**
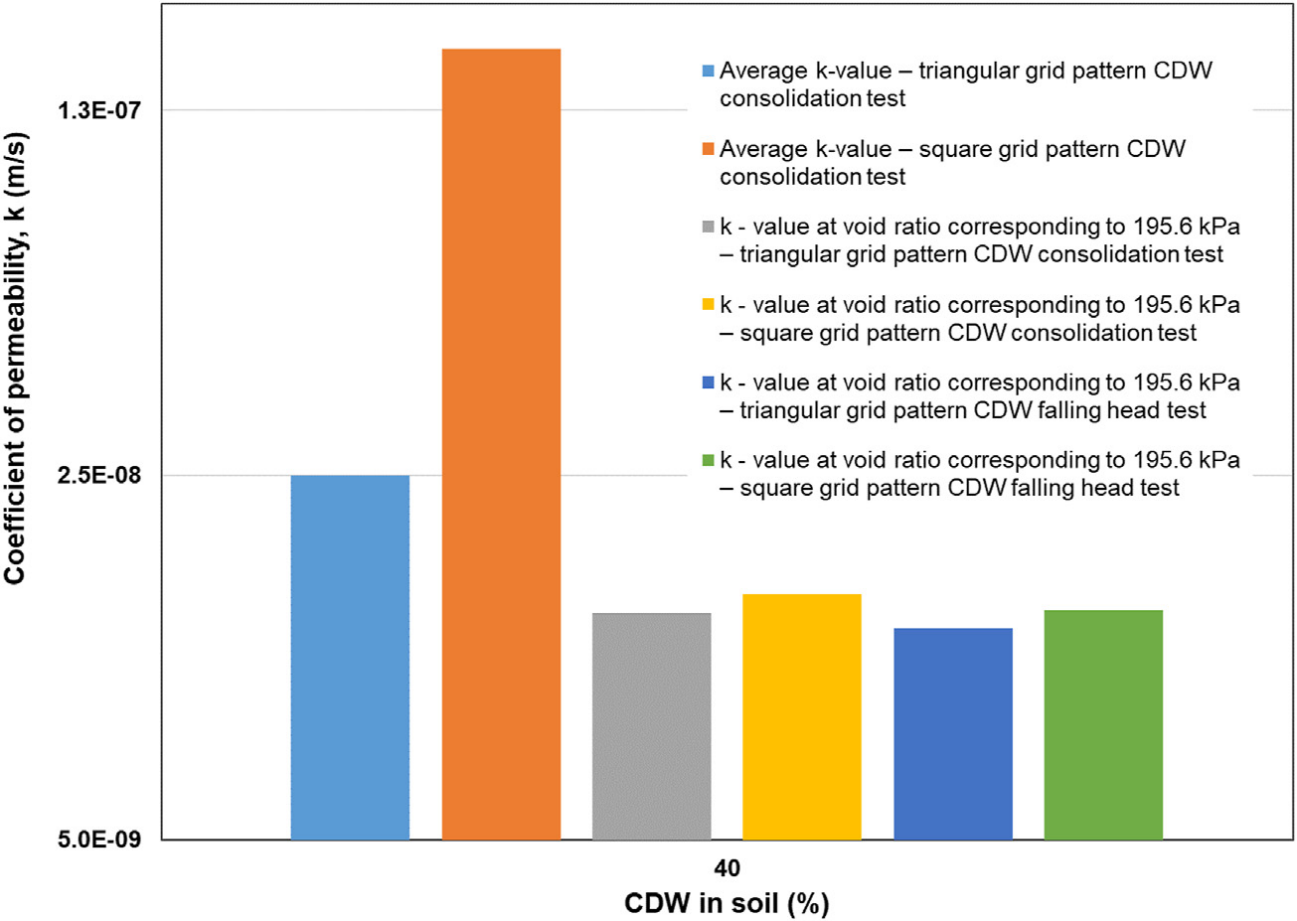
Coefficient of permeability vs. CDW (%) in CDW column inserted soil in triangular and square grid patterns.

All the soil samples, including soil with CDW column in triangular and square grid patterns, were tested at the void found at the beginning of the final loading (195.61 kPa) step to determine the coefficient of permeability following the falling head method of ASTM D5856 (2015). The values of *k* were determined as 1.14E-09 m/s, 2.23E-09 m/s, 2.47E-09 m/s, 5.15E-09 m/s, 5.32E-09 m/s, 1.27E-08 m/s, and 1.38E-09 m/s for the original soil sample, soil with CDW 10%, soil with CDW 20%, soil with CDW 30%, soil with CDW 40%, soil sample with CDW column in a triangular grid pattern, and the soil sample with CDW column in square grid pattern, respectively. The values of *k* determined in falling head tests were similar to *k* found in the consolidation tests. Figures 9 and 10 describe the value of coefficient of permeability, *k* with respect to CDW percentages in soil, soil-CDW mixture, CDW column inserted soil for the average value of *k* after all the loading stages, the value of *k* at the void ratio corresponding to 195.6 kPa load in consolidation test, the value of *k* at the void ratio corresponding to 195.6 kPa load in falling head test. The comparison between the value of *k* obtained by consolidation test and the falling head test is shown in Table 4. As the samples were more consolidated and more voids were removed at the last step of incremental loading, the average value of the coefficient of permeability for different soil samples was found to be bigger than the k-value at the final stage of loading (195.6 kPa) (Figure 9). The average values of coefficient of permeability with the changes in CDW powder column in the soil mass in triangular and square grid patterns of different permeability measurements are presented in Figure 10, highlighting square grid pattern CDW column in the soil mass shows highest average *k*-value.

**Table 4:** Comparison of *k* value in between consolidation test and falling head test.

Consolidated soil type	Coefficient of permeability, k (m/s) at final stage void ratio	
	consolidation test	Falling head test
Original soil	1.08E-09	1.14E-09
Soil with 10% CDW	2.29E-09	2.23E-09
Soil with 20% CDW	2.52E-09	2.47E-09
Soil with 30% CDW	5.07E-09	5.15E-09
Soil with 40% CDW	5.56E-09	5.32E-09
CDW Column in triangular pattern	1.36E-08	1.27E-08
CDW Column in square pattern	1.48E-08	1.38E-08

#### 3.3.5 Pre-consolidation pressure

**Figure 11:**
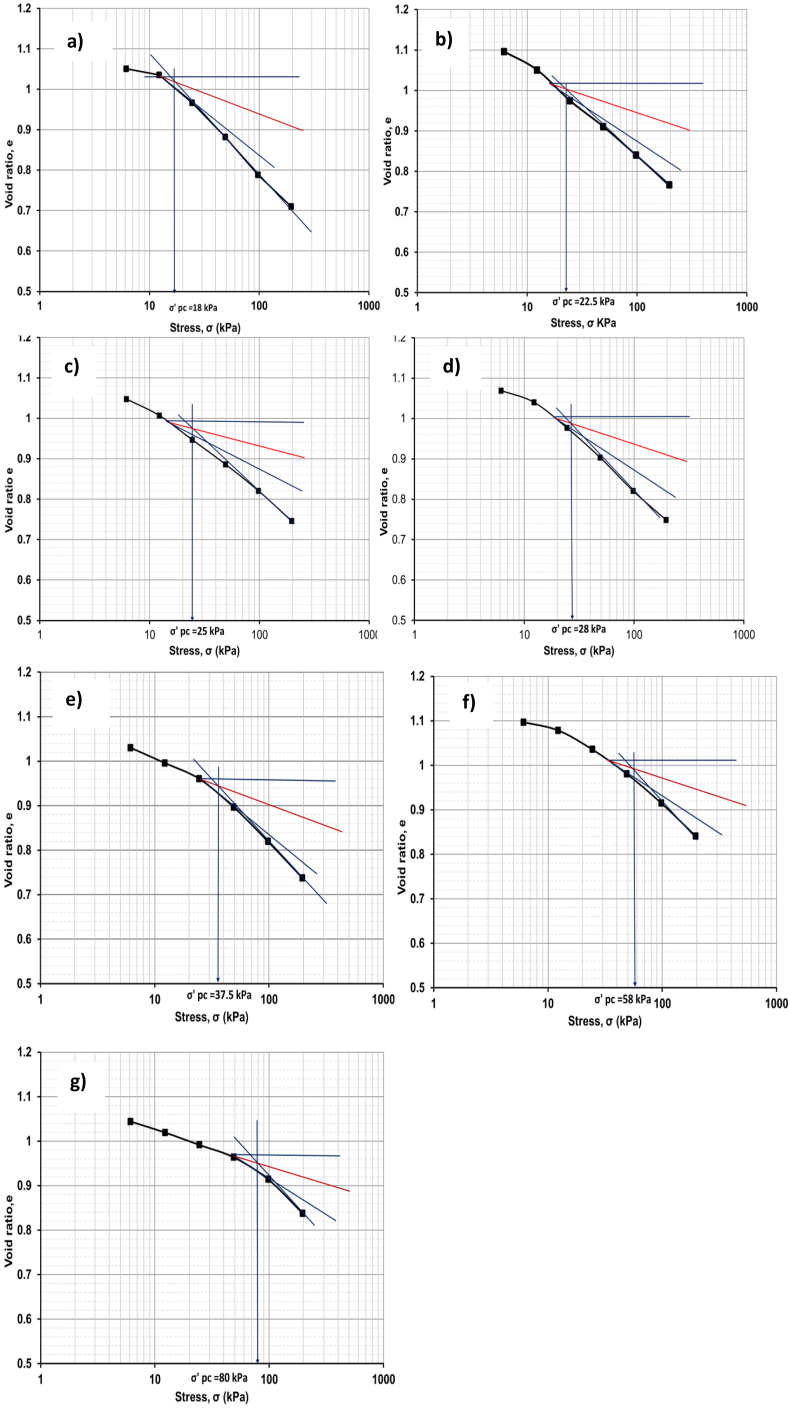
Pre-consolidation pressure a) Original soil sample b) soil with CDW10% c) soil with CDW 20% d) soil with CDW30% e) soil with CDW40% f) soil with CDW column in triangular grid pattern g) soil with CDW column in square grid pattern.

Pre-consolidation pressure, *σ’*_*pc*_ of the soil samples, was determined according to the Casagrande graphical method (Dias and Pierce, 1995). Figure 11 shows the void ratio, *e* vs. log *σ* graph, also expressed as a compression curve for the soil samples tested in this research. The pre-consolidation pressure, *σ’*_*pc*_, is calculated from the void ratio, *e* vs. log *σ*, graph bisecting by following Casagrande's graphical method for different soil samples. For the original soil sample pre-consolidation pressure is 18 kPa. And just after adding 10% CDW, *σ’*_*pc*_ becomes slightly larger as 22.5 kPa and found as 25 kPa for the soil with 20% CDW. Further increment in CDW as 30% gave the more increased *σ’*_*pc*_ value as 28 kPa, and the soil sample containing 40% CDW resulted in a maximum *σ’*_*pc*_ value of 37.5 kPa among the soil-CDW mixtures. The soil sample with CDW powder columns in triangular and square grid patterns experienced a more *σ’*_*pc*_ value than the uniformly mixed soil-CDW mixtures. The soil samples with CDW columns in triangular and square grid patterns have an upgraded pre-consolidation pressure of 58 kPa and 80 kPa, respectively, which are supported by Umar and Sadrekarimi (2016). As the CDW powder columns are inserted into the soil, it becomes easier to consolidate the soil and drain the water to have the required settlement in a short time.

#### 3.3.6 Justification of the findings using direct shear test and SEM results

##### 3.3.6.1 Direct shear test results

**Figure 12:**
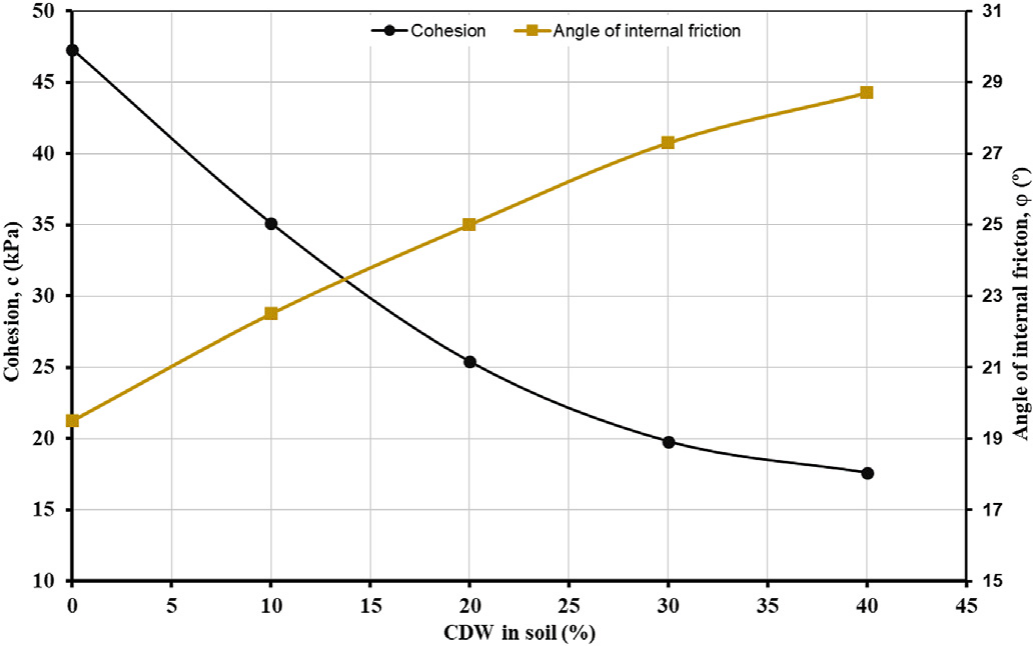
Cohesion and angle of internal friction of soil-CDW mixtures.

Direct shear tests on disturbed original soil and soil-CDW mixtures were carried out in the direct shear box of size 60 × 60 × 20 mm following ASTM D3080 (2011). The samples were prepared at their optimum moisture content and compacted state in the mold. The applied stresses on the specimens were 50, 100, and 200 kPa. The specimens were consolidated upon the application of vertical load for 24 h with inundation in the water bath of test assembly to the specimens saturated during testing. The test specimens were sheared at a slow rate of 0.01 mm/min to attain a drained condition. The specimens were sheared up to 14 cm to mobilize the shear strength completely and to achieve the peak shear strength. The cohesion and angle of internal friction of the original soil were found to be 47.3 kPa and 19.5° (Figure 12). With the addition of different percentages of CDW in the soil mass, the cohesion of the mixtures increased, and the angle of internal friction decreased, justifying the results of consolidation tests discussed before. Figure 12 also highlights that 40% CDW is the optimal content in the soil, though the trend of the plots didn't flatten yet because CDW contain clay-sized cement and sand. The percentage of CDW in the current research is marginally higher than some previous researches because of the type and particles size of CDW and soil (Jayatheja et al., 2017, 2021; Suluguru et al., 2018, 2019).

##### 3.3.6.2 SEM discussions

**Figure 13:**
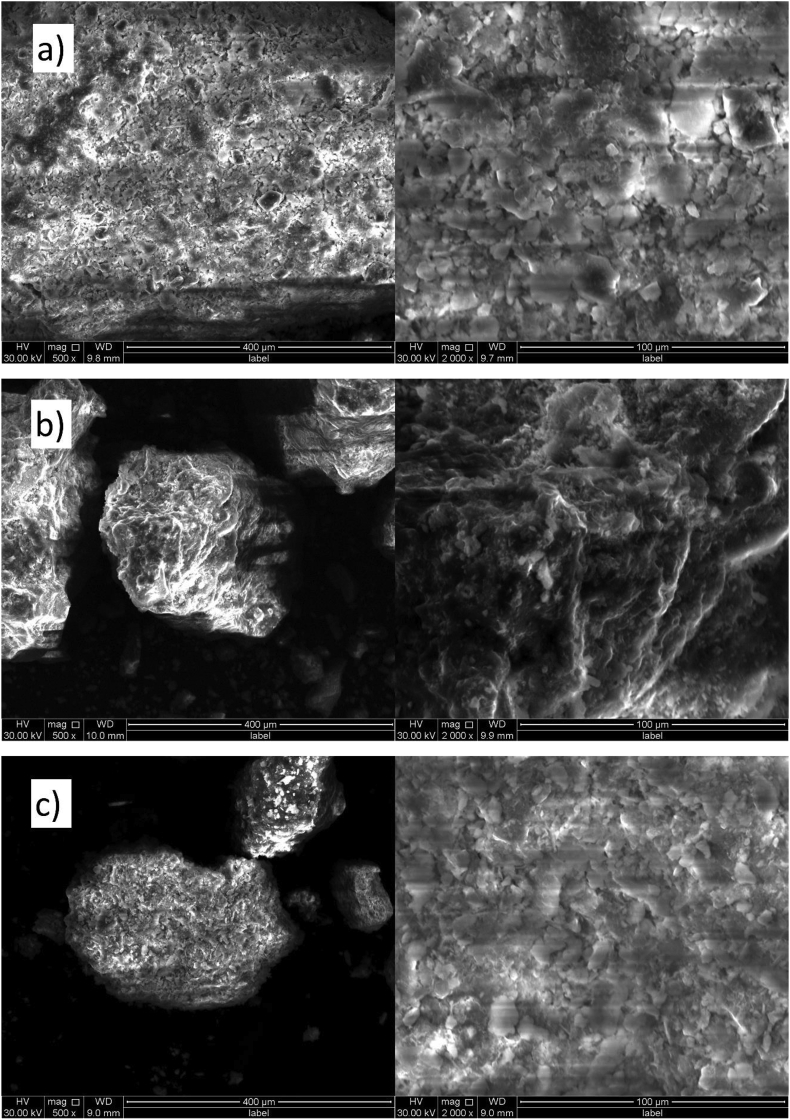
SEM images of (a) original soil sample, (b) CDW, and (c) soil +40% CDW.

**Figure 14:**
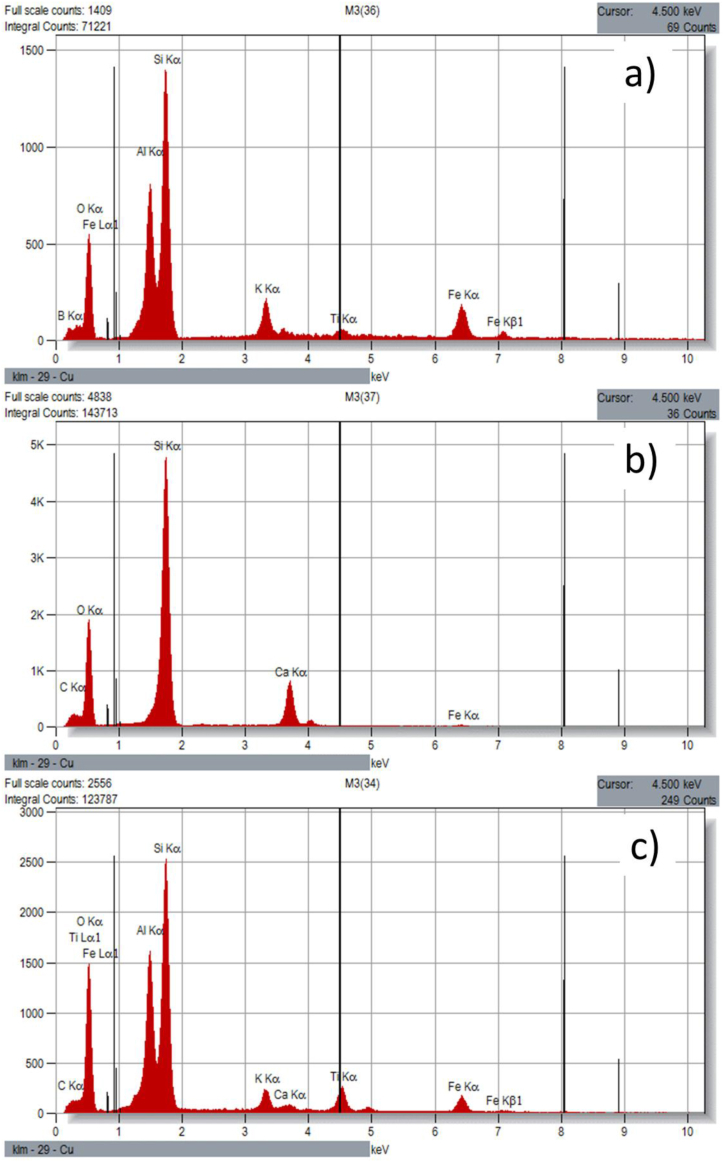
EDS images of (a) original soil sample, (b) CDW, and (c) soil +40% CDW.

The scanning electron microscope (SEM) images of original soil sample, construction and demolition waste (CDW) powder and soil +40% CDW are shown in Figure 13 (a), (b), and (c). It is seen that the original soil sample has a rough surface with irregular particles and voids while CDW powder is a bit regular and shiny but the surface is uneven. And the sample with 40% CDW powder also looks similar to the original soil sample but has reduced voids in the surface and looks denser. In the CDW stabilized soil sample, soil and CDW powder bond is clearly noticed. Again, in Figure 14 (a), (b), and (c) the energy dispersive spectroscopy (EDS) image of original soil sample, CDW, and soil with 40% CDW are shown. Although SEM image of original soil sample and soil +40% CDW looks similar, spectral image in Figure 14(c) reveal the differences between these two samples. The EDS quantitative results are shown in Table 5 gives the elemental data of the samples. The original soil sample contained B, O, Al, Si, k, Ti, Fe where O and Si occupied the major portion. In CDW powder Fe, Si, O are common as soil and also O and Si has the major portion of total amount while C and Ca are additional. The soil with 40% CDW contained C, O, Al, Si, K, Ca, Ti, and Fe where Al, Si, Ca and O are cementitious/hydrated matter that took the role to the reaction and made the soil more resistant to load and reduced settlement (Artuso and Lukiantchuki, 2019).

**Table 5:** SEM-EDS corresponding data from quantitative EDS results.

Original soil sample							
Element Line	Net Counts	Int. Cps/nA	Z	Weight %	Norm. Wt.%	Atom %	Formula
**B K**	331	0.12	0.934	13.94	13.94	24.97	B
**O K**	4916	1.781	0.943	37.36	37.36	45.2	O
**Al K**	8095	2.933	1.049	10.79	10.79	7.74	Al
**Si K**	17574	6.367	1.023	24.27	24.27	16.73	Si
**K K**	2748	0.996	1.085	3.81	3.81	1.88	K
**Ti K**	573	0.208	1.166	1.06	1.06	0.43	Ti
**Fe K**	3353	1.215	1.174	8.78	8.78	3.05	Fe
**Total**				100	100	100	
Construction and demolition waste (CDW)							

**C K**	2655	0.962	0.926	11	11	16.65	C
**O K**	18437	6.68	0.974	55.36	55.36	62.94	O
**Si K**	60350	21.866	1.057	26.77	26.77	17.33	Si
**Ca K**	12090	4.38	1.097	6.47	6.47	2.94	Ca
**Fe K**	402	0.146	1.213	0.4	0.4	0.13	Fe
**Total**				100	100	100	
Soil+ 40% CDW							

**Line**	**Counts**	**Cps/nA**			**Wt.%**		
**C K**	1720	0.623	0.912	9.57	9.57	15.21	C
**O K**	14117	5.115	0.959	50.02	50.02	59.66	O
**Al K**	16144	5.849	1.067	10.39	10.39	7.35	Al
**Si K**	31549	11.431	1.041	20.72	20.72	14.08	Si
**K K**	2966	1.075	1.104	1.92	1.92	0.94	K
**Ca K**	701	0.254	1.081	0.47	0.47	0.22	Ca
**Ti K**	3744	1.357	1.186	3.27	3.27	1.3	Ti
**Fe K**	2917	1.057	1.194	3.63	3.63	1.24	Fe
**Total**				100	100	100	

After all the discussion above, it is clear that the optimum CDW powder content is found as 40% in this research. Although different researchers got different optimal CDW, the researchers state that being optimum material largely depends on CDW sizes and types, soil type, mineralogical properties and soil-aggregate interaction physics. The CDW particle size used in this research is fine-grained, which is close to the soil particle size used here, having good mixing ability and showed good consolidation properties.

## 4 Summary and conclusion

The research was conducted to investigate the compressibility and hydraulic behavior of the clay soil in different ratios of construction and demolition waste (CDW). The Atterberg limit, consolidation settlement, coefficient of consolidation, compression index, permeability, and pre-consolidation pressure of the original soil, soil-CDW mixtures, and soil with circular CDW columns in triangular and square grid patterns were assessed. The following general observations can be drawn based on the experimental analyses conducted.

⁃ The liquid limit and plastic limit of the soil reduces with the increase of CDW percentages in the soil mix. Soil sample containing 40% CDW shows the lower values of liquid limit and plastic limit.
⁃ A significant reduction in the settlement is found with the increase of CDW content. The original soil sample has the maximum settlement, and the soil sample with circular CDW column in triangular and square grid patterns show the minimum settlement, which is about 30% less than the original soil sample.
⁃ The coefficient of consolidation increases with the increase in CDW percentage and maximum for soil with CDW column in a square grid pattern. The compression index decreases with the increase of CDW content and minimum for soil with CDW column in a triangular grid pattern.
⁃ The coefficient of permeability increases with the increase in CDW percentage and becomes maximum for square grid pattern CDW column inserted soil. The values of *k* increase by about 36-times and 5-times for the soil with the circular CDW column in square and triangular grid patterns, respectively.
⁃ The pre-consolidation pressure increases with the increase in CDW percentages, producing 3-times and 4-times higher values for the soil with CDW columns in a triangular and square grid pattern, respectively.
⁃ According to the outcomes of the research, recycled CDW can be applied to improve soft soil to construct a shallow foundation. Powder CDW column can be implemented as an alternative to the natural resources (like sand) commonly used to improve weak clay soil.

## Declarations

### Author contribution statement

Shriful Islam: Conceived and designed the experiments; Analyzed and interpreted the data; Contributed reagents, materials, analysis tools or data; Wrote the paper.

Junaidul Islam: Performed the experiments; Analyzed and interpreted the data; Contributed reagents, materials, analysis tools or data; Wrote the paper.

Nur Md. Robiul Hoque: Conceived and designed the experiments; Analyzed and interpreted the data; Wrote the paper.

### Funding statement

This research did not receive any specific grant from funding agencies in the public, commercial, or not-for-profit sectors.

### Data availability statement

Data included in article/supp. material/referenced in article.

### Declaration of interest’s statement

The authors declare no conflict of interest.

### Additional information

No additional information is available for this paper.
